# Comprehensive Genomic Profiling of EBV-Positive Diffuse Large B-cell Lymphoma and the Expression and Clinicopathological Correlations of Some Related Genes

**DOI:** 10.3389/fonc.2019.00683

**Published:** 2019-07-25

**Authors:** Yangying Zhou, Zhijie Xu, Wei Lin, Yumei Duan, Can Lu, Wei Liu, Weiping Su, Yuanliang Yan, Huan Liu, Li Liu, Meizuo Zhong, Jianhua Zhou, Hong Zhu

**Affiliations:** ^1^Department of Oncology, Xiangya Hospital, Central South University, Changsha, China; ^2^Department of Pathology, Xiangya Hospital, Central South University, Changsha, China; ^3^Department of Orthopedics, Xiangya Hospital, Central South University, Changsha, China; ^4^Department of Pharmacy, Xiangya Hospital, Central South University, Changsha, China

**Keywords:** EBV-positive diffuse large B-cell lymphoma (EBV+ DLBCL), next-generation sequencing, fluorescence *in situ* hybridizations, immunohistochemistry, MYC, KMT2D

## Abstract

Epstein-Barr virus (EBV)-positive diffuse large B-cell lymphoma (EBV+ DLBCL) is a rare type of lymphoma with a high incidence in elderly patients, poor drug response, and unfavorable prognosis. Despite advances in genomic profiling and precision medicine in DLBCL, EBV+ DLBCL remain poorly characterized and understood. We include 236 DLBCL patients for EBV-encoded mRNA (EBER) *in situ* hybridization detection and analyzed 9 EBV+ and 6 EBV negative cases by next-generation sequencing (NGS). We then performed fluorescence *in situ* hybridization (FISH) and immunohistochemistry (IHC) to analyze chromosome rearrangements and gene expressions in 22 EBV+ and 30 EBV negative cases. The EBER results showed a 9.3% (22/236) positive rate. The NGS results revealed recurrent alterations in *MYC* and *RHOA*, components of apoptosis and NF-κB pathways. The most frequently mutated genes in EBV+ DLBCL were *MYC* (3/9; 33.3%), *RHOA* (3/9; 33.3%), *PIM1* (2/9; 22.2%), *MEF2B* (2/9; 22.2%), *MYD88* (2/9; 22.2%), and *CD79B* (2/9; 22.2%) compared with *KMT2D* (4/6; 66.7%), *CREBBP* (3/6; 50.0%), *PIM1* (2/6; 33.3%), *TNFAIP3* (2/6; 33.3%), and *BCL2* (2/6; 33.3%) in EBV-negative DLBCL. *MYC* and *KMT2D* alterations stood out the most differently mutated genes between the two groups. FISH detection displayed a lower rearrangement rate in EBV+ cohort. Furthermore, KMT2D expression was highly expressed and associated with poor survival in both cohorts. MYC was only overexpressed and related to an inferior prognosis in the EBV+ DLBCL cohort. In summary, we depicted a distinct mutation profile for EBV+ and EBV-negative DLBCL and validated the differential expression of KMT2D and MYC with potential prognostic influence, thereby providing new perspectives into the pathogenesis and precision medicine of DLBCL.

## Introduction

Epstein–Barr virus-positive (EBV+) diffuse large B cell lymphoma (DLBCL), not otherwise specified (NOS), is a new and rare type of DLBCL according to the 2016 WHO lymphoma classification, with clinically highly aggressiveness and no history of immunosuppression. Compared with the former classification as “EBV-positive diffuse large B-cell lymphoma of the elderly” in 2008 WHO, it removed the age denominator, as it was also noted in young adults and children (1–3). EBV+ DLBCL was observed worldwide, with a distinct incidence between 2 and 15% of all DLBCLs. It is most prevalent in Asian populations (8–15%), followed by Latin America (7%), and is rare in Western countries (<5%) (4–6). EBV+ DLBCL has an obvious age inclination, occurring mostly in people over 50 years, with a median age of 71 and male pre-dominance (4, 7, 8). Patients frequently present with extranodal involvement, and more than half had advanced disease with high International Prognostic Index (IPI) scores. These patients also showed a poor response to the conventional R-CHOP (anti-CD20 monoclonal antibody-rituximab, combined with cyclophosphamide, vincristine doxorubicin, and prednisone) regimen, with an inferior survival rate of ~24 months (2, 9, 10).

Epstein-Barr virus (EBV) is an enveloped, double-stranded virus which belongs to the Herpesviridae family with demonstrated B-cell lymphotropic and oncovirus properties. The EBV genome encodes a series of products that interact with various anti-apoptotic molecules, signal transducers, and cytokines, thus promoting EBV infection, immortalization, and transformation. (2, 11). Almost 90% of humans worldwide are exposed to EBV or harbor lifelong latent infection. EBV infection is associated with several lymphoid malignancies, including extranodal NK/T-cell lymphoma (ENKTCL), Burkitt lymphoma (BL), Hodgkin lymphoma (HL), and angioimmunoblastic T-cell lymphoma (AITL) (12–14). The incidence of developing an EBV-associated lymphoma is low and is influenced by race, geography, heredity, immunity, and infection cofactors (14, 15).

Because of the rarity and variable distribution of EBV+ DLBCL, its pathogenesis is still unclear, especially in the field of genomics in China. This has also impeded the treatment of EBV+ DLBCL. With the advancement of next-generation sequencing (NGS) technology, molecular composition and function of various malignancies (including EBV+DLBCL) have been comprehensively and effectively analyzed. Some studies have demonstrated that EBV+ DLBCL is characterized by the activation of the JAK-STAT and NF-κB pathways (16, 17). While some mutations can be detected in *CD79B* and *CARD11*, no *MYD88* mutations were detectable (18). Copy number alterations (CNAs) and gene expression profiles showed that relatively few genomic alterations were found in EBV+ DLBCL compared with EBV-negative DLBCL (19). However, the genomic profile of EBV+ DLBCL is still unclear.

In this study, we performed NGS to investigate genomic alterations in EBV+ DLBCL and EBV-negative DLBCL samples. We then performed fluorescence *in situ* hybridization (FISH) for cytogenetic studies, detected some significant genes by immunohistochemistry (IHC) and correlated their expression with the clinicopathological features of DLBCL patients to provide new insights into the pathogenesis and potential prognostic factors or treatment opportunities for EBV+ DLBCL.

## Materials and Methods

### Case Selection

A cohort of 236 DLBCL specimens was obtained from Xiangya Hospital of Central South University from May 2013 to November 2016. All cases underwent *in situ* hybridization for EBV-encoded mRNA (EBER) and fulfilled the World Health Organization criteria for diagnosis. Other criteria included the absence of other immunodeficiency causes and the diagnosis of EBV-positive diffuse large B-cell lymphoma, EBV-positive Hodgkin's lymphoma, EBV-positive pleomorphic lymphoproliferative disorders or any previous lymphoma-related treatment. Avoiding DNA degradation and impacting the sequencing quality, we only choose the formalin-fixed paraffin-embedded (FFPE) samples in the latest 2 years for further NGS detection. Only 10 EBER-positive samples from the recent 2 years, which had complete specimens of more than 50% tumor cells and <10% necrosis tissues, were used for DNA extraction and next-generation sequencing. For the rest of the EBV-negative DLBCLs, we randomly chose 30 cases for comparison, which came from the different diagnostic year and qualified all the requirements above. For NGS, we had six EBER negative cases from the latest 2 years which matched the age, gender, Ann Arbor stage, and IPI score with 9 EBV+DLBCL patients, and they all belong to the 30 EBV-negative DLBCL cases. All paraffin-embedded specimens were collected following the ethical standards of the human experimental committee (institutional and national).

Clinical information, including gender, age, LDH level, B symptoms, Ann Arbor stage, anatomic locations, International Prognostic Index (IPI) score, and survival time, was collected. Complete clinical information and follow-up data were collected and all patients were followed up from the date of diagnosis to November 2018. All patients were treated with R-CHOP-based chemotherapy and basic supportive regimen. Imaging strategies were used to evaluate the treatment response. Progression-free survival (PFS) and overall survival (OS) were calculated for survival analysis.

### *In situ* Hybridization for EBV

*In situ* hybridization for EBERs was performed on deparaffinized 4 mm-thick, FFPE tissue sections using fluorescein-binding oligonucleotide probes and a Dako Detection Kit for EBV-encoded small RNA (EBER, Dako Cytomation, Denmark). The slides were counterstained with hematoxylin and fixed in Faramount (Dako Cytomation). All slides were viewed under a standard bright field microscope by two independent pathologists. Positive staining was recognized by a yellow to the brown color in the nucleus. All cases with >80% of EBER-positive malignant cells were considered EBV-positive (1).

### DNA Isolation

Considering DNA degradation of the FFPE tissue specimens, 10 EBV+DLBCL and six EBV-negative DLBCL FFPE tissue specimens in the latest 2 years were obtained from archive materials. The FFPE material contains at least 50% of tumor cells and <10% necrotic tissue. DNA was extracted from a number of wax roll samples to ensure the total DNA content of cancer cells was more than 100 ng. FFPE samples were deparaffinized using 300 μl of xylene three times for 3 min each. Xylene was rehydrated with graded alcohol and washed three times in 1 mM EDTA (pH 8.0) for 3 min. The pelleted samples were incubated overnight with 20 ml proteinase K and 150 ml buffer ATL. QIAamp DNA-FFPE Tissue Kit (Qiagen, Hilden, Germany) was used for DNA obtaining. The Qubit fluorometer 2 (Life Technologies, Darmstadt, Germany) was used for DNA concentration measurement. A sufficient amount of DNA was isolated from all archived FFPE samples for further analysis. One patient with EBV+DLBCL was from further analysis due to poor DNA quality.

### Next-Generation Sequencing

In our study, we used the lymphoma-related, 64-gene panel for next-generation sequencing (Burning Rock Biomedical Company, Guangzhou, China). All genes were related to lymphoma pathogenesis and possible targeted therapy (Supplementary Table 1). All the genes exon regions and part of intron regions were analyzed using probe hybridization enrichment method. According to the manufacturer's instructions, about 200 ng DNA was used for library preparation and sequencing on the Illumina Miseq system (Illumina Inc., San Diego, USA). The Agilent High Sensitivity DNA Kit (Agilent, Santa Clara, USA) was used for generating library size and quality.

### Fluorescent *in situ* Hybridization

Interphase fluorescence *in situ* hybridization (FISH) was performed using 4 μm FFPE tissue sections. Slides were deparaffinated with graded alcohol and deionized water and treated with protease on VP 2000 (Abbott Molecular, IL, USA) for 25 min. After treatment, 5–10 μl of *c-MYC, BCL6*, and *BCL2* break-apart probes (Abbott Molecular, IL, USA) were added and sealed with neutral resin. The slides were then placed overnight in a 37-degree hybridization chamber and washed with SSC/NP 40 and dehydrated with gradient ethanol. The slides were counterstained with 5–10 μl DAPI and allowed to cool in the freezer for 15 min. Two different pathologists observed a total of 200 cells, each reading 100 cells. If more than 10% of the tumor cells showed a single fusion and/or separate green and red signals, the *c-MYC, BCL2*, and *BCL6* rearrangements were considered positive.

### Immunohistochemistry

We detected the expression of KMT2D and MYC by streptavidin-peroxidase conjugated assay. The staining was performed following the manufacturer's instructions for the staining kit. The resected 4 μm thick tissue sections were dewaxed and hydrated. Heat antigen recovery was applied in ethylenediaminetetraacetic acid (EDTA, pH 9.0) for 20 min, followed by 3% hydrogen peroxide and blocked by serum for 30 min, respectively. All slides were incubated at 4°C overnight with primary antibody, anti-KMT2D rabbit polyclonal antibody (Millipore, ABE206, USA, 1:500 dilution) and anti-MYC Y69 rabbit monoclonal antibody (Abcam, ab32072, USA, 1:100 dilution). The biotin-bound secondary antibody was added and incubated for 30 min at 37°C. Subsequently, diaminobenzidin (DAB) was added and incubated for 1–2 min for a coloration time.

KMT2D and MYC immunohistochemistry expression were evaluated based on the intensity and percentage of stains using a semiquantitative system (20, 21): negative, weak, moderate and strong. For the intensity score, 0 = no yellow signal, 1 = light yellow signal; 2 = yellow signal; and 3 = brown signal. For percentage score, 0 = entirely negative staining (0%); 1 = weak staining (<20% of tumor cells); 2 = moderate staining (20–50% of tumor cells); and 3 = strong staining (>50% of tumor nuclei). The total score was the addition of these two scores: 0 score is negative (–); 1–2 points are weak positive (+); 3–4 points are moderate positive; 5–6 points are the strong positive. Negative and weak positive were considered to be the low expression; moderate, and strong positive was regarded as high expression. All slides were evaluated in a double-blind manner by two pathologists. If there was a disagreement, consensus scores were determined by a third experienced pathologist. Appropriate negative and positive controls were included in the IHC assay.

### Bioinformatics Analysis

After sequencing, the filtered raw data were harvested using FastQC. Then, high- quality reads were compared with the human genome (GRCH38, UCSC hg38) by using the Burrows Wheeler Aligner software program. Indel realignments on the reads overlapping target regions were captured by GATK's Realigner Target Creator and Indel Realigner tools (22, 23). Generally, mutations occurred in at least 20% of the reads that were accepted; otherwise, IGV was manually utilized for the alteration frequencies between 5 and 20%. The DAVID Bioinformatics Resources 6.8 web server was applied for Gene Ontology (GO) and Kyoto Encyclopedia of Genes and Genomes (KEGG) pathways enrichment analysis. The *P*-value was adjusted by using the Benjamini and Hochberg algorithm, and *P* < 0.05 was considered to be significantly enriched (24, 25). GeneCards and TCGA databases were searched for gene annotations and outcome comparison.

### Statistical Analysis

SPSS 24.0 (SPSS Inc., Chicago, USA) was used to analyze the characteristics of the clinicopathological information, and χ^2^ and Fisher's exact tests were used to calculate the *P* value. The chi-square test and Fisher's exact test were used to analyze the clinical characteristics between gene mutation or protein expression in DLBCL patients. Comparing the differential immunohistochemistry expression of KMT2D and MYC was performed using the Wilcoxon test. Correlation analysis of KMT2D and MYC expression was using Spearman rank correlation analysis. Multivariate prognostic correlation analysis was using Cox proportional hazard regression model. Survival analysis was calculated according to the Kaplan–Meyer method. All trials were conducted with bilateral 95% confidence intervals (CIs). If *P* < 0.05, it was considered statistically significant.

## Results

### Patients and Clinical Information

From the files of our institutions, during the period from May 2013 to November 2016, EBV infection was detected in more than 80% of neoplastic cells in 22 of 236 (9.3%) diffuse large B cell lymphomas by EBER *in situ* hybridization (Supplementary Figure 1). Of 22 EBV+ DLBCL patients, the male to female ratio was 1.4:1, and there were 14 (63.6%) patients more than 60 years old. Ten patients (45.5%) had an increased level of lactate dehydrogenase (LDH), and only 6 (27.3%) suffered a B symptom. According to the Visco-Young algorithm (26), six had the germinal center B cell (GCB) subtype, and 16 patients (72.7%) had the activated B cell (ABC) subtype. There were 15 patients (68.2%) with an advanced stage based on the Ann Arbor stage. For the anatomic sites of tumors, 10 patients (45.5%) had extranodal involvement, including spleen (9.1%), gastric (9.1%), soft tissue (9.1%), colon (9.1%), and tonsil (9.1%). Regarding the IPI score, six patients (27.3%) were the low-risk (0–1 point), nine (40.9%) were the medium-risk (2–3 points), and seven (31.8%) were the high-risk (4–5 points). After R-CHOP-based chemotherapy, the median survival time was 23 months, ranging from 4 to 36 months. In comparison with randomly chosen 30 cases of EBV-negative DLBCL, patients with EBV+ DLBCL showed an elderly age distribution and more advanced Ann Arbor stage (*P* < 0.05). All the clinicopathological characteristics of 22 EBV+ DLBCL and 30 EBV-negative patients are summarized in Table 1.

**Table 1:** Clinicopathological features of EBV+/EBV– DLBCL patients.

**Characteristics**	**EBV(+)**	**EBV(–)**	***P***
	**(*N* = 22)**	**(*N* = 30)**	
Gender			0.947
Female	9(40.9%)	12(40.0%)	
Male	13(59.1%)	18(60.0%)	
Age			**0.03**
<60	8(36.4%)	20(66.7%)	
≥60	14(63.6%)	10(33.3%)	
LDH			0.093
Normal	12(54.5%)	23(76.7%)	
Elevated	10(45.5%)	7(23.3%)	
B symptoms			0.961
Absent	16(72.7%)	22(73.3%)	
Present	6(27.3%)	8(26.7%)	
Subtype			0.539
ABC	16(72.7%)	24(80.0%)	
GCB	6(27.3%)	6(20.0%)	
Ann Arbor Stage			**0.044**
I–II	7(31.8%)	18(60.0%)	
III–IV	15(68.2%)	12(40.0%)	
Location			0.523
Nodal	12(54.5%)	19(63.3%)	
Extranodal	10(45.5%)	11(36.7%)	
Spleen	2(9.1%)	3(10%)	
Soft tissue	2(9.1%)	1(3.3%)	
Gastric	2(9.1%)	3(10%)	
Colon	2(9.1%)	2(6.7%)	
Tonsil	2(9.1%)	1(3.3%)	
Nasal Cavity	0	1(3.3%)	
IPI score			0.984
Low-risk group	6(27.3%)	8(26.7%)	
Median-risk group	9(40.9%)	13(43.3%)	
High-risk group	7(31.8%)	9(30.0%)	
Survival Time			0.242
Median	23	29	
Range	4–36	4–51	

*ABC, activated B-cell-like type; GCB, germinal center B-cell-type; CNS, central nervous system; IPI, International Prognostic Index. The bold values showed P < 0.05*.

### Mutation Profile of EBV+ DLBCL

**Figure 1:**
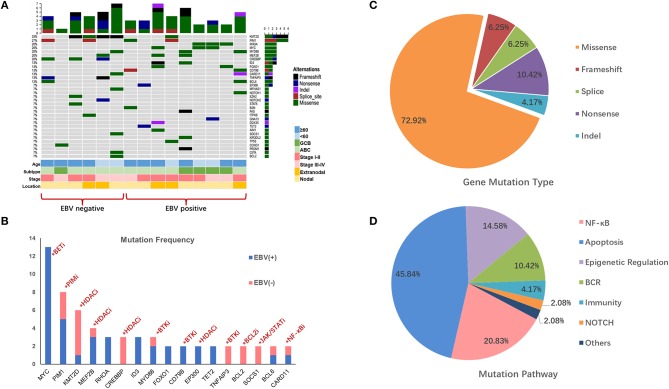
Mutation profile in EBV+ and EBV- negative DLBCL groups. **(A)** The distribution and frequency of genetic alterations in nine EBV+ DLBCL and six EBV- negative DLBCL. The types of mutation and different clinical factors are labeled in different colors. **(B)** Gene mutation with potential targeted therapy. The frequent mutation genes in EBV+ and EBV- negative DLBCL cohorts and the potential targets highlighted by indicating the possible targeted therapy. **(C)** Pie chart showing the percentages of different types of somatic mutation in EBV+ DLBCL. **(D)** Mutation pathway distribution among EBV+ DLBCL specimens. Different color represents diverse mutation pathways and percentages.

In this study, we detected and analyzed nine EBV+ DLBCL and six EBV-negative DLBCL patients using a lymphoma-related 64-gene panel for targeted sequencing. The clinicopathological features of 15 cases for sequencing are summarized in Supplementary Table 2. All genes exon regions and parts of intron regions were amplified. The average depth for sequencing was more than 1,000, representing the advantages and effectiveness of the high-throughput method. In total, 48 mutations and 22 diverse gene mutations were detected in EBV+ DLBCL cases. Of these, the most frequently altered genes in our patient groups were *MYC* (3/9; 33.3%), *RHOA* (3/9; 33.3%), *PIM1* (2/9; 22.2%), *MEF2B* (2/9; 22.2%), *MYD88* (2/9; 22.2%), and *CD79B* (2/9; 22.2%). Compared with EBV+ DLBCL, the recurrent mutation genes for EBV-negative DLBCL were *KMT2D* (4/6; 66.7%), *CREBBP* (3/6; 50.0%), *PIM1* (2/6; 33.3%), *TNFAIP3* (2/6; 33.3%), and *BCL2* (2/6; 33.3%). We found that *MYC* and *KMT2D* were the most differentially mutated genes between EBV+ and EBV-negative DLBCL (Figures 1A,B, Supplementary Table 3).

We further investigated the gene profiles between the EBV-positive DLBCL and EBV-negative DLBCL according to their different age, ABC or GCB, stages, and anatomic locations. We explored that with age over 60, *KMT2D, PIM1*, and *CREBBP* were frequently mutated in EBV-negative patients, while *MYD88, ID3*, and *FOXO1* highly altered in EBV+ DLBCL group. *PIM1* alterations were activated in ABC subtype in both EBV+ and EBV-negative groups, whereas *CREBBP* and *TNFAIP3* were more frequently mutated in EBV-negative group. Besides, with advanced stage, mutations of *MYC, MYD88, MEF2B*, and *ID3* were more commonly found in EBV+ DLBCL group. We also found that *RHOA* and *MYC* were pre-dominantly mutated in the EBV+ DLBCL cases with nodal invasive (Figure 1A).

The mutation types of genes in EBV+ DLBCL samples can be divided into five categories, including missense mutation, non-sense mutation, frameshift mutation, splicing mutation, and deletion mutation. Among them, missense mutation was the most common (72.92%) type, followed by non-sense mutations (10.42%), frameshift mutations (6.25%) and splice mutations (6.25%) (Figure 1C).

We further grouped the mutation genes into six specific pathways (Supplementary Table 4): apoptosis/cell cycle (*MYC, RHOA, FOXO1, GNA13*, and *BCL2*), NF-κB (*PIM1, CARD11, MYD88, PRDM1*), epigenetic regulation (*KMT2D, MEF2B, EP300*), B cell-receptor (*CD79B, ID3*), and NOTCH1 and immune-related (*B2M, KIR3DL2*) signaling pathways. From the results, the apoptosis/cell cycle signaling pathways were the most frequently mutated ones, taking up 48.83% of cases, followed by the NF-κB signaling pathway (20.83%), epigenetic regulation signaling pathways (14.58%) and B cell-receptor signaling pathway (10.42%) (Figure 1D).

**Figure 2:**
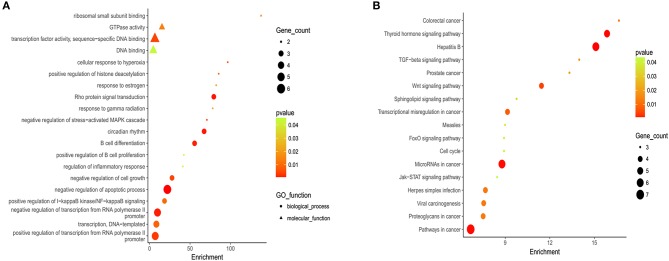
Gene ontology (GO) and Kyoto Encyclopedia of Genes and Genomes (KEGG) analysis of EBV+ DLBCL. **(A)** Bioinformatic analysis for the top 20 GO enrichment in EBV+ DLBCL. **(B)** Bioinformatic analysis for the top 15 KEGG pathway enrichment in EBV+ DLBCL. (The *P*-value denoted the significance of GO terms enrichment in the genes and was adjusted by using Benjamini and Hochberg method to avoid false positives, and *P* < 0.05 was considered as significantly enriched).

Gene Ontology (GO) and KEGG analyses are widely used in the field of bioinformatics. The top 20 most enriched categories in the Molecular Function (MF) and the Biological Process (BP) are shown in Figure 2A, and the top 15 essential pathways are shown in Figure 2B. Bioinformatics analysis demonstrated that the mutation genes of EBV+ DLBCL group were primarily participated in the biological behavior of cell regulation and involved in the process of cancer development and progression.

### Potential Clinical Influence of Mutated Genes

In EBV+ DLBCL patients, each mutated gene was analyzed and correlated with clinical features, including age, gender, B symptom, LDH level, origin subtype, Ann Arbor stage, and IPI score. We found that the *PIM1* mutation was related to LDH level (*P* = 0.048), and the *RHOA* variant was significantly associated with the cell of origin subtype in the patient (*P* = 0.048).

We further explored whether the most prevalent variants were related to clinical outcomes. Kaplan-Meier analyses were performed for the most common mutations using median progression-free survival (mPFS) and median overall survival (mOS) as readouts (Supplementary Tables 5, 6). The results demonstrated that patients harboring an *RHOA* mutation in their lymphoma showed a favorable PFS and OS (*P* < 0.05).

### Fluorescence *in situ* Hybridization for *MYC, BCL6* and *BCL2*

**Figure 3:**
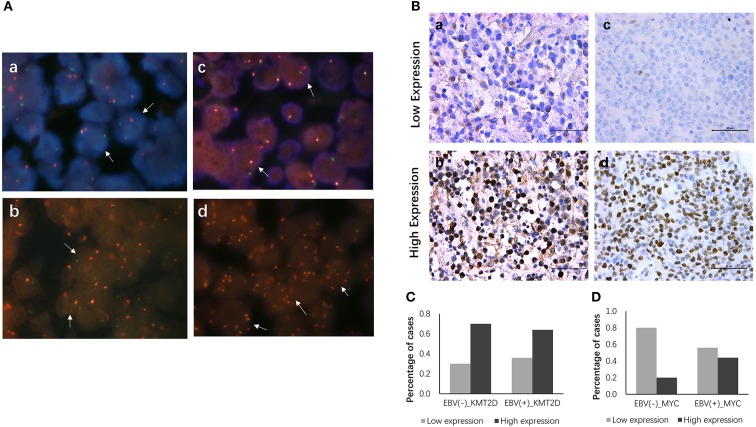
FISH and IHC staining of EBV+ and EBV negative DLBCL specimens. Example of FISH performed on EBV+ and EBV negative DLBCL **(A)**, FISH assay reveals rearrangement at the c-MYC (a), BCL2 (b) and BCL6 (c) loci using the dual-color break-apart rearrangement probes, break-apart signals in DLBCL nuclei are highlighted exemplarily by white arrows; increased copies of BCL6 by FISH (d). Immunohistochemistry staining for KMT2D and MYC in EBV+ and EBV negative DLBCL specimens **(B)**, (a) Low expression of KMT2D (IHC, ×400); (b) High expression of KMT2D (IHC, ×400); (c) Low expression of MYC (IHC, ×400); (d) High expression of MYC (IHC, ×400). **(C)** Bar chart showing the percentages of KMT2D expression in EBV+ and EBV- negative DLBCL cohorts. **(D)** Bar chart showing the percentages of MYC expression in EBV+ and EBV negative DLBCL cohorts.

All 22 EBV+ and 30 EBV-negative DLBCL samples were used for cytogenetic detection in the C-*MYC, BCL6*, and *BCL2* loci. After FISH analysis, 20 EBV+ and 26 EBV-negative DLBCL cases were satisfactory for evaluation. In EBV+ DLBCL patients, positive breaks in two of 20 cases (10.0%) of the *C-MYC* gene were detected. Three out of 20 cases (15.0%) showed positive *BCL6* rearrangement, and one had gain/amplification. No positive signal was found from the *BCL2* break-apart rearrangement probe. In EBV-negative cases, five of 26 cases (19.2%) had a positive split signal of the *C-MYC* gene, and two cases had gene amplification. Seven cases (26.9%) harbored a chromosomal breakage event affecting the *BCL6* locus, and three showed increased copies. *BCL2* was found to be split into only 2 (7.7%) cases, and none had *BCL2* amplification. Besides, only one case showed both *C-MYC* and *BCL6* rearrangements. Representative images are shown in Figure 3A.

### Immunohistochemical Expression of KMT2D and MYC

We then performed KMT2D and MYC immunohistochemistry in samples from 22 EBV+ DLBCL and 30 EBV-negative DLBCL patients. KMT2D was expressed in both the nucleus and cytoplasm. MYC staining was pre-dominantly nuclear with little cytoplasmic staining (Figure 3B). As we stated in the methods, negative and weak positive were considered the low expression, and moderate and high positive were regarded as high expression. In 22 EBV+ DLBCL patients, the high expression rates of KMT2D and MYC were 63.63% (14/22) and 45.45% (10/22), respectively. In 30 EBV- DLBCL patients, the high expression rates of KMT2D and MYC were 70.0% (21/30) and 20.0% (6/30), respectively. The high expression proportion of MYC in EBV+DLBCL and EBV-negative DLBCL patients was statistically significant (*P* < 0.05), while the expression of KMT2D was not statistically significant (*P* > 0.05) (Figures 3C,D, Table 2).

**Table 2:** The expression of KMT2D and MYC in EBV+/EBV– DLBCL patients.

**Histology**	**No**.	**KMT2D**			***P***	**MYC**			***P***
		**Low**	**High**	**High (%)**		**Low**	**High**	**High (%)**	
EBV+ DLBCL	22	8	14	63.63%	0.629	12	10	45.45%	**0.049**
EBV– DLBCL	30	9	21	70.00%		24	6	20.00%	

*The bold values showed P < 0.05*.

### Correlation of KMT2D and MYC Expression With Patients' Clinicopathological Features

We investigated the correlations of KMT2D and MYC expression with clinical features, including gender, age, LDH level, origin subtype, Ann Arbor stage, and IPI scores. In the EBV+ DLBCL cohort, the expression of KMT2D was related to the Ann Arbor stage and IPI scores (*P* = 0.002, and *P* = 0.019, respectively). The expression of MYC was related to the IPI score (*P* = 0.008). In the EBV-negative DLBCL group, the high expression of KMT2D was associated with a higher IPI score (*P* = 0.005), and the overexpression of MYC was also related to the advanced Ann Arbor stage (*P* = 0.026). However, we didn't find statistically significant correlations with patient gender, age, LDH level, B symptoms, and subtype (*P* > 0.05) (Table 3).

**Table 3:** Correlation of KMT2D and MYC expression with clinicopathological features.

**Characteristics**	**KMT2D**						**MYC**					
	**EBV(+) (*****N*** **=** **22)**			**EBV(–) (*****N*** **=** **30)**			**EBV(+) (*****N*** **=** **22)**			**EBV(–) (*****N*** **=** **30)**		
	**Low**	**High**	***P***	**Low**	**High**	***P***	**Low**	**High**	***P***	**Low**	**High**	***P***
Gender			0.806			0.745			0.429			0.709
Female	3	6		4	8		4	5		10	2	
Male	5	8		5	13		8	5		14	4	
Age			0.315			0.675			0.571			0.372
<60	4	4		7	13		5	3		17	3	
≥60	4	10		2	8		7	7		7	3	
LDH			0.746			0.393			0.639			0.29
Normal	4	8		8	15		6	6		17	6	
Elevated	4	6		1	6		6	4		7	0	
B symptoms			0.315			0.067			0.056			0.3
Absent	7	9		9	13		11	5		19	3	
Present	1	5		0	8		1	5		5	3	
Subtype			0.624			0.329			0.162			0.302
ABC	5	11		6	18		7	9		18	6	
GCB	3	3		3	3		5	1		6	0	
Ann Arbor Stage			**0.002**			0.249			0.074			**0.026**
I–II	6	1		7	11		6	1		17	1	
III–IV	2	13		2	10		6	9		7	5	
IPI score			**0.019**			**0.005**			**0.008**			0.087
Low-risk group	5	1		6	2		6	0		7	1	
Median-risk group	2	7		2	11		5	4		12	1	
High-risk group	1	6		1	8		1	6		5	4	

*P was calculated by χ^2^ and Fisher's exact tests. The bold values showed P < 0.05*.

We further examined the Kaplan-Meier survival analysis among our cohorts. The correlation of clinicopathological characteristics and the expression of KMT2D and MYC with survival time are summarized in Table 4. In EBV+ DLBCL, the median OS for all patients was 23 months, ranging from 4 to 36 months. We found that the age and IPI score were significant predictors for OS (*P* < 0.05), while other factors, such as gender, LDH level, cell of origin subtype, and Ann Arbor stage were not statistically significant (*P* > 0.05). According to the Kaplan-Meier analysis, the overexpression of KMT2D and MYC were both inferior prognostic factors in EBV+ DLBCL patients, with an mOS of 17 months vs. 29 months (*P* = 0.012) and 16 months vs. 29 months (*P* = 0.001), respectively. In EBV-negative DLBCL groups, the median OS of all cohorts was 29 months, ranging from 4 to 51 months. Only the IPI score and KMT2D expression significantly contributed to the mOS (*P* < 0.05) (Supplementary Figure 2). Multivariate Cox regression model analysis did not reach statistical significance between the two groups.

**Table 4:** Association of mOS and clinicopathological features.

**Characteristics**	**EBV(+) (*****N*** **=** **22)**		**EBV(–) (*****N*** **=** **30)**	
	**mOS (months)**	***P***	**mOS (months)**	***P***
Gender		0.612		0.96
Female	21		28	
Male	24		29	
Age		**0.002**		0.237
<60	31		35	
≥60	17		29	
LDH		0.373		0.843
Normal	25		29	
Elevated	18		28	
B symptoms		0.21		0.33
Absent	24		30	
Present	17		24	
Subtype		0.46		0.056
ABC	21		26	
GCB	22		35	
Ann Arbor Stage		0.057		0.067
I–II	31		34	
III–IV	18		23	
IPI score		**0.006**		**0.001**
Low-risk group	31		38	
Median-risk group	21		28	
High-risk group	13		17	
KMT2D		**0.012**		**0.002**
Low expression	29		41	
High expression	17		23	
MYC		**0.001**		0.073
Low expression	29		31	
High expression	16		20	

*P was calculated by χ^2^ and Fisher's exact tests. The bold values showed P < 0.05*.

## Discussion

Recently, Schmitz et al. redefined the genetic landscape of DLBCL for four subtypes (27) and Bjoern Chapuy et al. have also described the genetic profile of DLBCL for five subsets (28). The new classifications represented different genotypes, epigenetic, and clinical characteristics, which may provide a potential pathological basis for the precision medicine in DLBCL. Besides, it is essential to effectively combine advanced techniques with clinical practices and develop new targeted therapies, especially in the era of individualized medicine. In our study, we detected gene mutation status in both EBV+ and EBV- negative DLBCL patients using NGS technology. We revealed a variant mutation profile for DLBCL patients and discovered the phenomenon of multi-locus gene mutation, which may contribute to future pathogenesis researches and provide new concepts for the diagnosis and treatment of EBV+ DLBCL.

In our cohort, EBV infection was detected in 9.3% (22/236) of DLBCL by EBER *in situ* hybridization. The incidence was in accord with the range among Asian populations in previous studies (4, 29). We further found that there was a significantly different distribution of patients' age and Ann Arbor stage between EBV+ and EBV-negative DLBCL (*P* < 0.05), which confirmed that EBV+ DLBCL existed an elderly age-dominated feature and suggested a more aggressive pathogenic process (29). However, we did not reach statistical significance in patients' overall survival time between the two groups (*P* > 0.05), which was limited by the relatively small sample size and a short follow-up period.

In our study, we used NGS to detect 64 genes related to lymphoma pathogenesis and targeted therapy. In all nine EBV+ DLBCL samples, 48 mutations and 22 different gene mutations were detected. Overall, more than 65% of alterations were discovered in apoptosis and NF-κB pathway components, including *MYC* and *RHOA*. The *MYC* and *RHOA* mutations played the leading role in the variants, followed by *PIM1*, MEF2B, *MYD88*, and *CD79B*. For EBV-negative DLBCL patients, *KMT2D, CREBBP, PIM1, TNFAIP3*, and *BCL2* were the most frequently altered genes. Comparing these two types, *MYC* and *KMT2D* stood out as the most distinctive mutation genes between EBV+ and EBV-negative DLBCL. This novel finding implied a possible potential role in the development of DLBCL and indicated heterogeneous tumor characteristics that may be associated with tumor invasion, metastasis, and relapse. We also explored diverse gene profiles according to different clinical features in EBV+ and EBV-negative groups and need to be further investigated. In addition, we discovered different mutation profiles with other studies (17–19, 30, 31), which may be associated with the analytical methods, regional differences, sample types, different races, or tumor heterogeneities.

Diffuse large B cell lymphoma (DLBCL) is an aggressive non-Hodgkin lymphoma with distinct heterogeneity, morphological features, molecular subtypes, immunophenotypes, and clinical manifestations and prognosis. Previous researches have shown that EBV+ DLBCL was frequently activated by the NF-kB and JAK-STAT signaling pathways (16, 32). Our findings were consistent with previous studies but also revealed some peculiarities with the pre-dominant apoptotic/cell cycle signaling pathway (45.84% of total variance), followed by the NF-κB (20.83%), epigenetically related (14.58%), and B cell receptor signaling pathways (10.42%). The diverse distribution of genes and associated signaling pathways may be correlated with different sequencing methods, as well as various racial and geographical distributions.

Some studies found that the mutation of *RHOA* was a genetic hallmark in AITL and peripheral T-cell lymphomas (PTCL), while the specific role of *RHOA* remained unknown (33–35). Our results showed that the RHOA mutation had a favorable prognosis for EBV+ DLBCL, which provided a new perspective for the research of *RHOA*. However, our predictive results were limited by the small sample size and required further large cohorts validations.

Recently, NGS-based technology has provided more evidence for accurate diagnosis, prognosis, and precise treatment. The lymphoma-related gene mutations might have an irreplaceable role in treatment options. The mutations in our cohort may act as alternative targetable treatments (Figure 1B, Supplementary Table 7). In DLBCL, small molecular inhibitors were widely used, which include Bruton's tyrosine kinase inhibitors (BTKi, ibrutinib), PIM kinase inhibitors (PIMi, SGI-1776), histone deacetylase inhibitors (HDACi, belinostat, vorinostat), PI3K inhibitors (PI3Ki, copanlisib, buparlisib), and protein kinase C inhibitors (PKCi, sotrastaurin) (30, 36–38). Several studies have demonstrated that DLBCLs with mutated MYD88 and/or CD79B were more sensitive to BTKi (37, 39). Besides, Vermaat et al. demonstrated a vital result of prominent mutual exclusivity between EBV infection, rearrangements, and MYD88/CD79B mutations, which established a distinct DLBCL subcategory in MYD88/CD79B-mutated tumors (40). In our cohort, *CD79B* and *MYD88* had a high mutation frequency, and one patient had *MYD88/CD79B* double mutation, which may be more acceptable to ibrutinib treatment, although it needs to be confirmed in future studies. Other studies have shown that *PIM1* variations are related to endogenous ibrutinib resistance and pan-PIM inhibitors combined with ibrutinib was more effective than ibrutinib monotherapy (41–43). In addition, alterations in *CARD11* and *TNFAIP3* inactivated the effects of ibrutinib and sotrastaurin, while the mutation of *CD79A/B* was related to high sensitivity to sotrastaurin (44, 45). Therefore, sequencing targeted gene mutation status may help in selecting qualified candidates for different targeted inhibitors, avoiding drug resistance and improving treatment efficacy.

We performed an interphase FISH analysis to sort for main chromosome translocations involving *C-MYC, BCL2*, and *BCL6*. Our results demonstrated a lower rearrangement rate in the EBV+ DLBCL cohort compared with EBV-negative DLBCL with 10 vs. 19.2%, 0 vs. 7.7%, 15 vs. 26.9%, of the *C-MYC, BCL2*, and *BCL6* loci, respectively. Although our analysis was limited to single loci, our results still consistent with the notion that EBV carcinogenesis may decrease additional chromosomal changes commonly found in EBV-negative DLBCL (3, 11, 16).

Lysine(K)-specific methyltransferase 2D (*KMT2D*), encodes a histone methyltransferase that targets histone H3 lysine 4 (H3K4) and regulates epigenetic transcription (46). Mutations have been reported in 89% of follicular lymphomas (FL), 50% of primary central nervous system lymphoma (PCNSL), 20–30% of DLBCL, 15% of splenic marginal zone lymphomas (MZL), and 10–15% of mantle cell lymphomas (MCL) (47–51). In our cohort, there was an obvious mutation difference between EBV+ and EBV-negative DLBCL with a frequency of 11.1 vs. 66.7%. This indicated that *KMT2D* played a vital role in the tumorigenesis and development of DLBCL. Besides, targeting epigenetic alterations is being widely developed, notably using histone deacetylase (HDAC) and DNA methyltransferase (DNMT) inhibitors (52). Previous studies have shown that in DLBCL, using HDAC inhibitors could re-establish acetylation levels of mutated *CREBPP* and *EP300*, which laid a foundation for further targeted therapy (53). The first-in-class EZH2 inhibitor, tazemetostat, demonstrated enhanced clinical activity in mutant follicular lymphoma, and DLBCL patients (54). Moreover, applying HDAC inhibitors or a combination of histone methyltransferase with BCL2 inhibitors was considered as a possible choice in both *BCL2* and *KMT2D* alterations (55). In our study, we included *KMT2D, CREBBP, EZH2, EP300, MEF2B*, and *TET2 in* our lymphoma panel. The mutation of these genes may all be related to potential targeted medicine and may become a new therapeutic option.

Our study detected the expression of KMT2D between EBV+ and EBV-negative DLBCL. Our results demonstrated that KMT2D was highly expressed in both EBV+ and EBV-negative DLBCL, with 63.63% (14/22) and 70.0% (21/30), respectively. There was no significantly different correlation between these two types (*P* > 0.05). The expression of KMT2D was correlated with Ann Arbor stage in EBV+ DLBCL and IPI score in both EBV+ and EBV-negative DLBCL (*P* < 0.05), indicating that KMT2D may participate in the growth and progression in DLBCL. However, no statistical significant correlation was reached for KMT2D expression with patients' gender, age, B symptom, LDH level, and cell of origin subtypes. Furthermore, high expression of KMT2D brought an unfavorable mOS compared with low expression groups in both EBV+ and EBV-negative DLBCL (*P* < 0.05). Our findings were similar to those in primary gastrointestinal diffuse large B cell lymphoma (PGI-DLBCL) and breast and colon cancers (56, 57), implying that KMT2D may act as a prognostic biomarker for DLBCL patients.

*MYC* acts as a proto-oncogene and encodes a multifunctional and pleiotropic transcription factor that plays a role in cell cycle progression, apoptosis, metabolism, protein biosynthesis, and cellular transformation (58, 59). MYC oncoprotein family had three members (c-MYC, l-MYC, and n-MYC) known to play an indispensable role in the pathogenesis of numerous human malignancies, and ~15% of human genes are considered to be regulated by the MYC protein (60, 61). Studies have shown that *MYC* gene alterations are present in nearly 100% of Burkitt lymphoma cases (62–64) and a minority of DLBCL (3–16%) cases (62, 65). The presence of mutations in DLBCL is related to inferior drug response and an unfavorable prognosis (66, 67). In this study, *MYC* alteration was the most frequently mutated gene in EBV+ DLBCL but not in EBV-negative DLBCL, suggesting an essential role in EBV+ DLBCL tumorigenesis and development. Furthermore, previous studies showed that bromodomain and extraterminal domain inhibitor (BETi) had potent antagonism to *MYC* transcriptional activity and protein expression and exhibited antitumor activity in various hematological malignancies, including DLBCL (68–70). The combination of BET inhibitors and BCL2 inhibitors decreased drug resistance in *MYC*-related lymphomas (71, 72). Our results also offered essential insight into clinical decision-making of EBV+ DLBCL patients.

We also explored the expression of MYC between EBV+ and EBV-negative DLBCL and analyzed the relationship between MYC and clinicopathological characteristics. Our results showed a higher ratio of overexpression in EBV+ DLBCL compared with EBV-negative DLBCL in 45.45% (10/22) vs. 20.0% (6/30) (*P* = 0.049). The differential expression of MYC between EBV+ and EBV-negative DLBCL confirmed the aggressive nature of EBV+ DLBCL and implied the significant role of MYC in the occurrence and progression of DLBCL. The expression of MYC was correlated with the IPI score in EBV+ DLBCL patients and Ann Arbor stage in EBV-negative DLBCL cohorts, which implied MYC might be parcipated in the tumorigenesis and progression of DLBCL. We also discovered that high expression of MYC was related to an inferior overall survival in EBV+ DLBCL patients (16 vs. 29 months, *P* = 0.001), but not in the EBV-negative DLBCL cohort (*P* > 0.05). Our results were in accordance with previous studies in PCNSL, AITL, and PTCL (73, 74), indicating the predictive treatment effects and prognosis of MYC in EBV+ DLBCL patients.

Next-generation sequencing (NGS) is becoming more readily available in current academic research but has not been fully applied in traditional clinical settings. Our research uncovered an interrelationship between these gene mutations and oncogenic signaling pathways, suggesting testable therapeutic interventions. However, our study was limited by a small sample size, short follow-up period and retrospective nature. In the era of precision medicine, the selection of DLBCL therapies based on individual genetic alterations is crucial. Under the guidance of new techniques and concepts, we prospect for accelerating and creating new and comprehensive molecular researches and related targeted treatment for aggressive cancers.

In summary, we identified a differential molecular signature with highly recurrent genetic lesions in apoptosis and NF-κB pathways, *MYC*, and *RHOA* in EBV+ DLBCL compared with EBV-negative DLBCL. FISH results displayed a lower rearrangement rate of *C-MYC, BCL6*, and *BCL2* in the EBV+ cohort. Furthermore, KMT2D expression was highly expressed and related to inferior survival in both EBV+ and EBV-negative DLBCL. MYC was overexpressed and related to an inferior prognosis in EBV+ DLBCL.

## Data Availability

The raw data supporting the conclusions of this manuscript will be made available by the authors, without undue reservation, to any qualified researcher.

## Ethics Statement

In this study, all procedures were approved by the Ethics Review Board of Xiangya Hospital, Central South University (Changsha, China), and gained the written consent of all patients.

## Author Contributions

HZ, JZ, WLiu, and MZ designed and supervised the study and critically revised the manuscript. YZ and ZX performed the experiments and acquired result data. YD, WLin, and CL helped to perform the partial experiments. HL, LL, YY, and WS collected the patient samples and information. YZ and CL performed the EBV-encoded mRNA *in situ* hybridization. YZ and YD performed the immunohistochemistry. YZ, WLin, and CL performed the fluorescence *in situ* hybridization. ZX helped to review the statistical analysis. YZ drafted the manuscript. All authors read and approved the final version of the manuscript.

### Conflict of Interest Statement

The authors declare that the research was conducted in the absence of any commercial or financial relationships that could be construed as a potential conflict of interest.

## Supplementary Material

Supplementary Figure 1 EBER *in situ* hybridization detection in 236 DLBCL specimens. **(A)** Negative expression of EBER in DLBCL (ISH, x100); **(B)** Negative expression of EBER in DLBCL (ISH, × 400); **(C)** Positive expression of EBER in DLBCL (ISH, × 100); **(D)** Positive expression of EBER in DLBCL (ISH, × 100).

Supplementary Figure 2 Kaplan-Meier analysis of EBV± DLBCL patients with differential KMT2D and MYC expression. **(A)** Overall survival for KMT2D high and low expression in EBV+ DLBCL patients (*n* = 22, *P* = 0.012). **(B)** Overall survival for MYC high and low expression in EBV+ DLBCL patients (*n* = 22, *P* = 0.001). **(C)** Overall survival for KMT2D high and low expression in EBV- DLBCL patients (*n* = 30, *P* = 0.001). **(D)** Overall survival for MYC high and low expression in EBV- DLBCL patients (*n* = 30, *P* = 0.073).
