# Supplementary figures and images for: Comprehensive Genomic Profiling of EBV-Positive Diffuse Large B-cell Lymphoma and the Expression and Clinicopathological Correlations of Some Related Genes
Source: Front Oncol. 2019 Jul 25;9:683. doi: 10.3389/fonc.2019.00683 (PMC6669985; doi:10.3389/fonc.2019.00683)

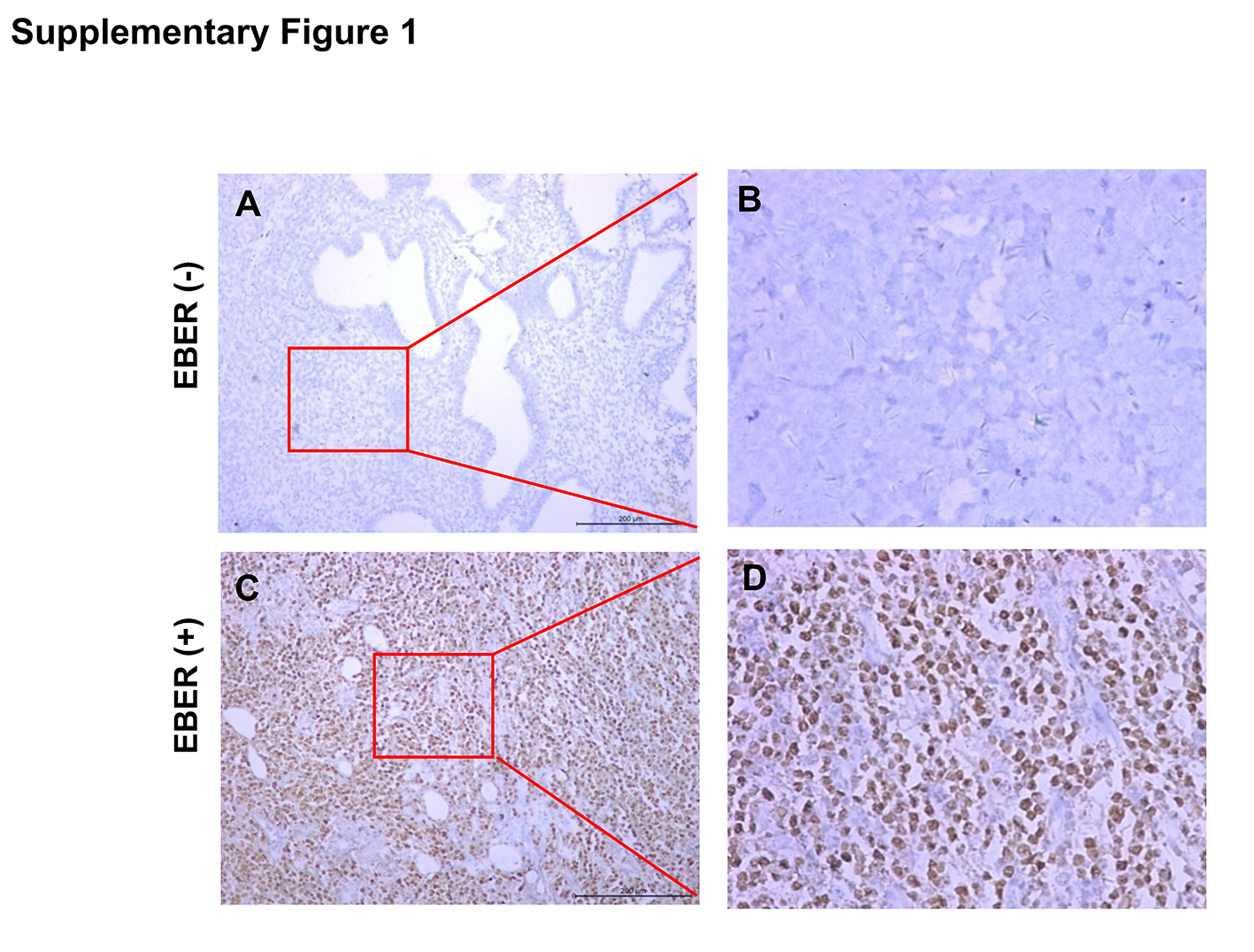

Supplement: Supplementary Figure 1 — EBER in situ hybridization detection in 236 DLBCL specimens. (A) Negative expression of EBER in DLBCL (ISH, x100); (B) Negative expression of EBER in DLBCL (ISH, × 400); (C) Positive expression of EBER in DLBCL (ISH, × 100); (D) Positive expression of EBER in DLBCL (ISH, × 100). [file Image_1.TIF]

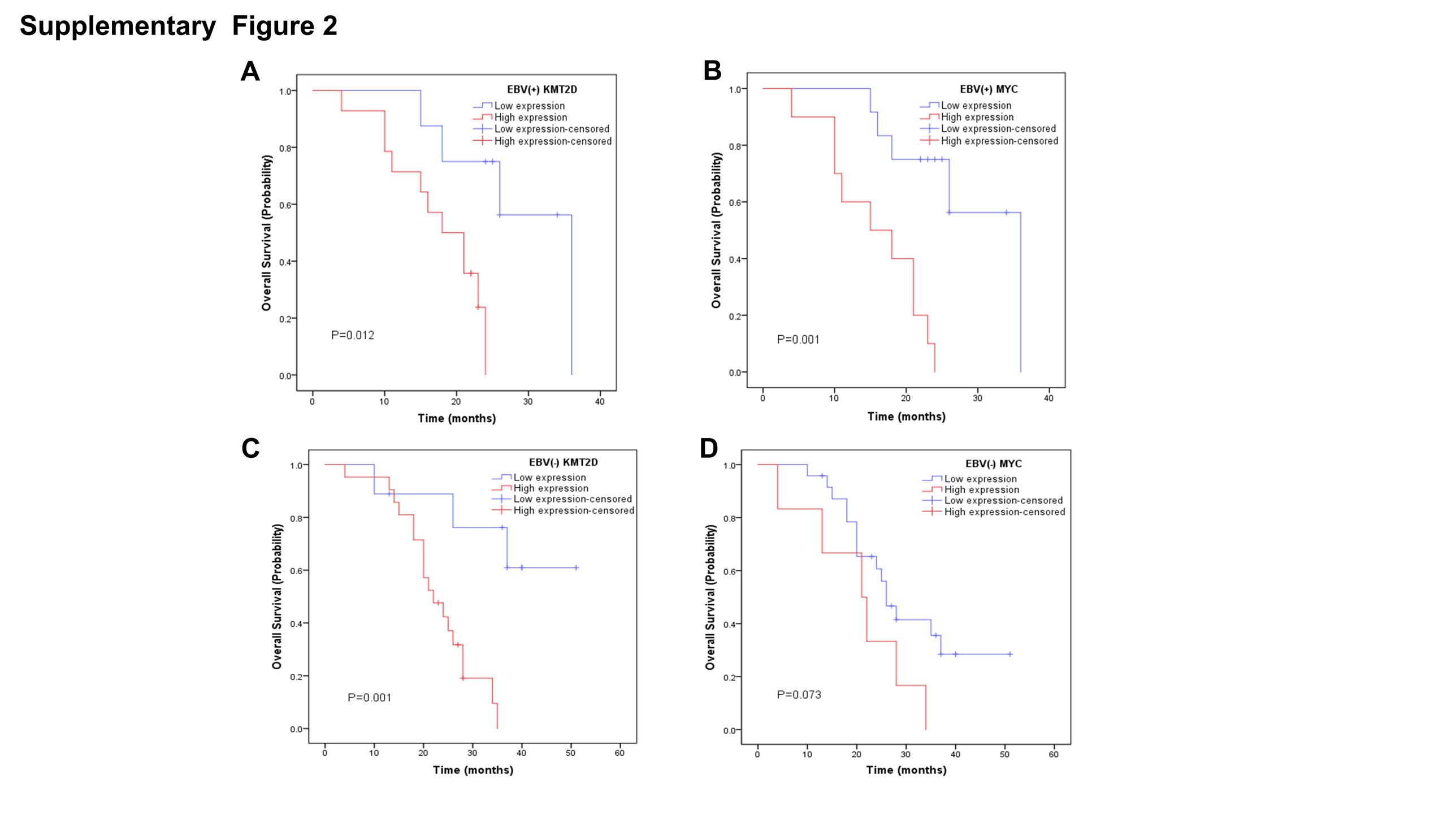

Supplement: Supplementary Figure 2 — Kaplan-Meier analysis of EBV± DLBCL patients with differential KMT2D and MYC expression. (A) Overall survival for KMT2D high and low expression in EBV+ DLBCL patients (n = 22, P = 0.012). (B) Overall survival for MYC high and low expression in EBV+ DLBCL patients (n = 22, P = 0.001). (C) Overall survival for KMT2D high and low expression in EBV- DLBCL patients (n = 30, P = 0.001). (D) Overall survival for MYC high and low expression in EBV- DLBCL patients (n = 30, P = 0.073). [file Image_2.TIF]
